# Human liver organoid derived intra-hepatic bile duct cells support SARS-CoV-2 infection and replication

**DOI:** 10.1038/s41598-022-09306-6

**Published:** 2022-03-30

**Authors:** Vincent Chi-Hang Lui, Kenrie Pui-Yan Hui, Rosanna Ottakandathil Babu, Haibing Yue, Patrick Ho-Yu Chung, Paul Kwong-Hang Tam, Michael Chi-Wai Chan, Kenneth Kak-Yuen Wong

**Affiliations:** 1 Department of Surgery, Li Ka Shing Faculty of Medicine, The University of Hong Kong, Pofulam Road, Pok Fu Lam, Hong Kong; 2 School of Public Health, Li Ka Shing Faculty of Medicine, The University of Hong Kong, Sassoon Road, Pok Fu Lam, Hong Kong; 3 Dr. Li Dak-Sum Research Centre, The University of Hong Kong – Karolinska Institutet Collaboration in Regenerative Medicine, The University of Hong Kong, Pok Fu Lam, Hong Kong; 4 Present Address: MRC WIMM Centre For Computational Biology, Medical Research Council (MRC) Weatherall Institute of Molecular Medicine (WIMM), John Radcliffe Hospital, University of Oxford, Oxford, UK

**Keywords:** Hepatology, Virology

## Abstract

Although the main route of infection for severe acute respiratory syndrome coronavirus 2 (SARS-CoV-2) is the respiratory tract, liver injury is also commonly seen in many patients, as evidenced by deranged parenchymal liver enzymes. Furthermore, the severity of liver damage has been shown to correlate with higher mortality. Overall, the mechanism behind the liver injury remains unclear. We showed in this study that intra-hepatic bile duct cells could be grown using a human liver organoid platform. The cholangiocytes were not only susceptible to SARS-CoV-2 infection, they also supported efficient viral replication. We also showed that SARS-CoV-2 replication was much higher than SARS-CoV. Our findings suggested direct cytopathic viral damage being a mechanism for SARS-CoV-2 liver injury.

## Introduction

Coronavirus disease-2019 (COVID-19) is caused by severe acute respiratory syndrome coronavirus 2 (SARS-CoV-2) and has rapidly become a worldwide pandemic. As of February 2022, there have been 402,044,502 confirmed cases of COVID-19 and 5,770,023 deaths worldwide^1^.

SARS-CoV-2 is a single-strand positive-sense RNA virus, belongs to the beta coronavirus family, which enters cells through the Angiotensin Converting Enzyme 2 (ACE2) receptor^2^. SARS-CoV-2 interacts with the host cells by first attaching its spike protein to ACE2 on host cells, and then gains entry through hemagglutinin cleavage by host cell protease Transmembrane protease, serine 2 (TMPRSS2)^3,4^. Human-to-human transmission for SARS-CoV-2 is efficient. While the respiratory tract is a common route of disease transmission^5,6^, the gastrointestinal tract has also been shown as another possible route of viral transmission^7–9^. Understanding the cellular/tissue tropism and the route of infection of SARS-CoV-2 virus is essential in overall patient management and infection control.

Liver damage is often identified as a typical occurrence in COVID-19 patients, and 58–78% of COVID-19 patients were shown to exhibit various degrees of liver injury^10^. Some COVID-19 patients have elevated levels of liver enzymes such as aspartate amino-transferase (AST), alanine aminotransferase (ALT) levels, and gamma-glutamyl transferase (GGT), while some patients have higher overall bilirubin levels and lower serum albumin^11–13^. Indeed, elevated AST, ALT, and total bilirubin levels but lower serum albumin levels are correlated with higher death rate^6^, and have been found in the severe group of COVID-19 patients^14^. Moreover, activation of coagulation and fibrinolysis accompanied by thrombocytopenia were observed in severe COVID-19 cases^15^. Autopsy examinations of a small number of COVID-19 patients have provided first evidence of secondary liver injury^16,17^. Subsequent studies with larger series of COVD-19 patients also suggested virally mediated liver injury based on clinical and histologic observations and endothelial cells might also be affected^18,19^.

Liver damage can be aggravated by the increase of COVID-19 infection severity, which indicates that the degree of liver damage may serve as an indicator of COVID-19 progression. Nonetheless, the mechanism of liver injury is poorly understood and may be due to direct viral hepatitis, bystander systemic inflammatory response or complications of drug treatment^20^.

Angiotensin converting enzyme 2 (ACE2), the protein through which SARS-CoV-2 gains entry, is abundantly expressed on many cells, including hepatocytes, bile duct cells and liver endothelial cells. ACE2 expression levels in bile duct cells is higher than those in hepatocytes and is comparable with alveolar epithelial type II cells^21^. Given that bile duct cells play an important role in immune defence and liver regeneration, their impairment may serve as a major cause of virus-induced liver injury in COVID-19 patients^22^. Although typical coronavirus particles characterized by spiked structures in the cytoplasm of hepatocytes have been identified^23^, the susceptibility of bile duct cells to SARS-CoV-2 infection is yet to be confirmed.

An organoid is a miniaturized and simplified version of an organ produced in vitro in 3-dimension. It shows realistic micro-anatomy and retain the biology of individual tissues. Lung and gut organoids have been successfully used to demonstrate SARS-CoV-2 infection^24–26^. Recently, human liver ductal organoids were shown to express ACE2 and TMPRSS2, and were permissive to SARS-CoV-2 infection^27^. The extra-and intra-hepatic bile duct cells are derivatives of different progenitors, in that extra-hepatic ducts arise from a common SOX17 immuno-positive/PDX1 immuno-positive pancreatobiliary progenitor, while the intra-hepatic ducts arise from CK19 immuno-positive/AFP immuno-positive hepatoblasts^28^. Furthermore, the cellular properties and functions of extra- and intra-hepatic cholangiocytes (bile duct cells) are very different. It remains to be investigated if intra-hepatic bile ducts are susceptible to SARS-CoV-2 infection and support viral replication, which would help provide more insight into SARS-CoV-2 associated liver damage. Recently, we have shown that the human liver tissue organoids derived from EPCAM immuno-positive cells of liver biopsies are from the hepatoblast progenitor (CK19 immuno-positive) lineage rather than from the pancreatobiliary (extra-hepatic ducts) progenitor (SOX17 immuno-positive/PDX1 immuno-positive) lineage, and thus recapitulates the intra-hepatic cholangiocyte development^29^.

In this study, we utilized human liver tissue derived organoids from hepatoblast progenitor as an ex-vivo tool to investigate the infection, tropism and replication competence of SARS-CoV-2 on intra-hepatic bile ducts and compared these parameters with SARS-CoV infection. We showed that liver tissue derived organoids consisted of intra-hepatic cholangiocytes and hepatoblasts as major cell types, which expressed ACE2 and TMPRSS2 and could be rapidly infected by both SARS-CoV-2 and SARS-CoV. SARS-CoV-2 was shown to have a much higher replication rate than SARS-CoV.

## Materials and methods

### Liver tissues

Pediatric liver tissues were obtained from non-tumour margin of hepatoblastoma (HB). Liver biopsies were obtained during operations with full informed consent from parents or patients, and the study was approved by Hong Kong West Cluster-Hong Kong University Cluster Research Ethics Committee/Institutional Review Board (UW 16–052). All experiments were performed in accordance with relevant guidelines and regulations.

### Human pediatric liver organoid culture

Wedge liver biopsies (1–2 cm^3^) from the non-tumor margin of pediatric patients (*n* = 4) with hepatoblastoma (HB)). Liver tissues were minced in cold wash medium (Advanced DMEM/F12; 1% GlutaMAX; 1% FBS; 1% Penicillin/Streptomycin (P/S)) and digested in digestion medium (5 ml, Multi Tissue Dissociation Kit 1; Miltenyi Biotec Inc. CA, USA) in a gentleMACS-C Tube (Miltenyi Biotec Inc. CA, USA) on gentleMACS™ Octo Dissociator (Miltenyi Biotec Inc. CA, USA) using program 37 °C-Multi-A-01. After digestion and washing with cold wash medium (5 ml), the digested sample was passed through a 70 µm strainer and then through a 30 µm strainer before centrifugation (300 g; 10 min) to pellet the cells. Cell pellet was resuspended in 200 µl of MACS column buffer (PBS pH 7.2 + 0.5% BSA), and incubated with CD326 (EPCAM) microbeads (1 µl microbeads/2 × 10^6^ cells; 130–090-500; Miltenyi Biotec Inc. CA, USA) in the dark at 4 °C for 30 min. After incubation, column buffer (0.5 ml) was added and EpCAM positive cells were sorted on MS Column following the manufacturer’s protocol (Miltenyi Biotec Inc. CA, USA). The cells were pelleted (300 g; 10 min.) and resuspended in 200 µl of column buffer for cell counting. The cell pellet (approximately 1 × 10^5^ cells) was mixed with 50–60 µl of Matrigel (356,231; Corning Biocoat) and seeded per well of a prewarmed (37 °C) 4-well plate (176,740; Nunc; Thermo Fisher Scientific). After Matrigel had solidified, organoid medium (500 µl) was added to each well. Organoid medium was based on Advanced DMEM/F12 (Invitrogen) supplemented with Penicillin/Streptomycin (Invitrogen), GlutaMax (Invitrogen), 25 mM HEPES (Invitrogen), 1% N2 (GIBCO), 1% B27 (GIBCO), 1.25 mM Acetylcysteine (Sigma), 10 nM gastrin (G9145; Sigma), 50 ng/ml EGF (PMG8043; PeproTech), 100 ng/ml FGF10 (100-26-25UG; PeproTech), 25 ng/ml HGF (100-39-10UG; PeproTech), 10 mM Nicotinamide (Sigma-Aldrich), 5 µM A83.01 (Tocris), 10 µM Forskolin (Tocris), 500 ng/ml R-Spondin 1 (7150-RS-025; R&D), 100 ng/ml Noggin (250-38-20UG; Peprotech), 100 ng/ml Wnt3a (1324-WN-010; R&D) and 250 ng/ml Amphotericin B (#15290018; GIBCO). For the first six days of culture, 10 µM ROCK inhibitor Y-27632 (Tocris) was added. Medium was changed once every two days.

### Single cell RNA sequencing and data analysis

Single cells from organoids were prepared. The organoid medium along with matrigel containing organoids was transferred to a 15 ml tube, 1–2 ml cold Advanced DMEM/F12 was added and the mixture was incubated on ice for 10 min to dissolve matrigel before centrifugation (300 g; 5 min). The supernatant was aspirated until the organoid pellet remained. 1 ml 5X TrypLE Express (12,604,013; GIBCO) was added, mixed well and incubated at 37 °C for 5 min. 1 ml FBS was added to the mixture, which was pipetted up and down for 40–50 times with a small circumference opening glass pipette (diameter 0.3–0.5 mm) to efficiently dissociate organoids into single cells. 5–10 ml cold Advanced DMEM/F12 medium was added, and the suspension was passed through a 30 µm strainer before centrifugation (300 g; 5 min) at 4 °C. The supernatant was aspirated until only the pellet remained and the cells were counted following addition of 200 µl 10% FBS. The cells were then counted using Invitrogen Countess II FL Automated Cell Counter. Samples were prepared as outlined by the 10 × Genomics Single Cell 3’ v2 Reagent Kit user guide. The samples with more than 1 × 10^6^ cells/ml along with > 80% viability is then resuspended in 50–100ul, 10% FBS and are submitted for 10X Genomics single-cell sequencing at Centre for PanorOmic Sciences (CPOS)-HKU. Sample viability was assessed by counting Trypan Blue stained cells (viable cells were not stained with Trypan Blue, while dead cells were stained with Trypan Blue) (Thermo Fisher Scientific) on haemocytometer (Thermo Fisher Scientific). Following counting, the appropriate volume, ~ 33.8 µl of each sample were used for cell encapsulation for a target capture of 4000 cells. Sequencing run were carried out using Illumina NovaSeq 6000 for those sequencing libraries passing the QC with good cDNA yield. Single cell transcriptomic data have been uploaded to NCBI Sequence Read Archive (SRA) and can be found with accession number: PRJNA609259.

The CellRanger (10X Genomics) analysis pipeline was used to generate digital gene expression matrix (UMI counts per gene per cell) from sequencing data by aligning to the human genome. The raw gene expression matrix was filtered, normalized and clustered using standard Seurat package procedures 16^30^. The low quality cells were removed from the analysis using the following thresholds: cells with a very small library size or UMI counts per cell (nUMI > 1500), genes detected per cell (nGene > 1000), UMIs vs. genes detected (log10GenesPerUMI > 0.8), mitochondrial counts ratio (mitoRatio < 0.1). Cell-cycle phases were predicted using a function included in Seurat that scores each cell based on expression of canonical marker genes for S and G2/M phases, this was used to regress out the cell-cycle effects from the downstream analysis. Clusters were visualized using uniform manifold approximation and projection (UMAP) as implemented in Seurat. First 13 PCs were selected for UMAP based on the points where the principal components cumulatively contribute 90% of variation associated with entire data. The cell-type identities for each cluster in UMAP were determined using presence of known marker genes in each cluster.

### Virus stock preparation

A SARS-CoV-2 (BetaCoV/Hong Kong/VM20001061/2020, SCoV2) was isolated from a COVID-19 patient in Hong Kong in 2020. For comparison, we used a SARS-CoV (strain HK39849, SCoV) isolated from a hospitalized SARS patient in Hong Kong in 2003. SARS-CoV-2 and SARS-CoV viruses were propagated in Vero-E6 cells and virus stock was titrated to determine tissue culture infection dose 50% (TCID_50_) in Vero-E6 cells. The experiments were carried out in a Bio-safety level 3 (BSL-3) facility.

### Coronavirus infection

The 3D liver organoids were sheared mechanically using syringe to expose the apical surface to the virus inoculum. Around 100–200 organoids were infected with each coronavirus at MOI 0.1 for 1 h at 37 °C. The organoids were washed three times with culture medium, re-embedded in Matrigel at the same conditions with the same growth medium and incubated at 37 °C with 5% CO_2_. The viral titers in the culture supernatants were measured at 1, 24, 48, and 72 h after infection using the TCID_50_ assay in Vero-E6 cells. Organoids were fixed 48 and 72 h after infection in paraformaldehyde for immunofluorescent staining. Two replicates were maintained for each infection experiments on liver tissue-derived organoids of two HB patients. At least 150 organoids were used for each transfection experiments.

### Immunofluorescence

Organoids in matrigel (from 2 HB livers) were fixed in 4% paraformaldehyde (w/v) in PBS (phosphate-buffered saline, pH 7.2) for 48 h at 4 °C, dehydrated in graded series of alcohol, and cleared in xylene before being embedded in paraffin. Sections (6 µm in thickness) were prepared and mounted onto TESPA-coated microscope glass. Sections were dewaxed in xylene, hydrated in a graded series of alcohol and finally in distilled water. Antigen retrieval was performed by incubation of slides in Citrate buffer (pH 6.0) at 95 °C for 10 min. After blocking in PBS-T (PBS with 0.1% Triton) supplemented with 1% Bovine Serum Albumin for 1 h at room temperature, sections were incubated with antibody diluted in PBS-T/BSA for overnight at 4 °C. After washing in PBS-T, sections were incubated with fluorescent tagged secondary antibodies in PBS-T/BSA at 37 °C for 1 h. Details of the primary and secondary antibodies and their dilutions are shown in Table S1. After PBS-T washings, sections were mounted in DAPI-containing anti-fade mounting fluid. Images were taken with Nikon Eclipse 80i microscope mounted with a SPOT RT3 microscope digital camera under fluorescence illumination. Photos were compiled using Adobe Photoshop CS6.

To quantitate the percentages of infection of organoids by SARS Coronavirus, we examined immunofluorescence stained sections of infected organoid cultures, counted the total of organoids on the section and the number of organoids that showed immuno-positive staining of Coronavirus nucleocapsid protein. To quantitate the percentages of infected organoid cells per infected organoids, we counted the total of organoid cells of infected organoid and the number of organoid cells that showed immuno-positive staining of Coronavirus nucleocapsid protein (SCov-NP). Percentage of infection was determined by dividing the number of infected organoids/total number of organoids × 100% or by dividing the number of infected cells/total number of cells of infected organoid × 100%. Percentages were reported as the calculated mean and standard deviation of mean.

### Statistical analysis

Statistical analysis was done using GraphPad Prism software version 9. Experiments with the human organoids were performed independently in two different donors each with triplicate wells. Viral titers and area under the curve (AUC) derived from viral titers and mRNA expression were compared using one- or two-way ANOVA with Tukey’s multiple comparisons test. Mock infected organoids served as negative controls. Results shown in figures are the calculated mean and standard deviation of mean. Differences were considered significant at *p* < 0.05.

## Results

### Expression of ACE2 and TMPRSS2 in human liver organoids

**Figure 1:**
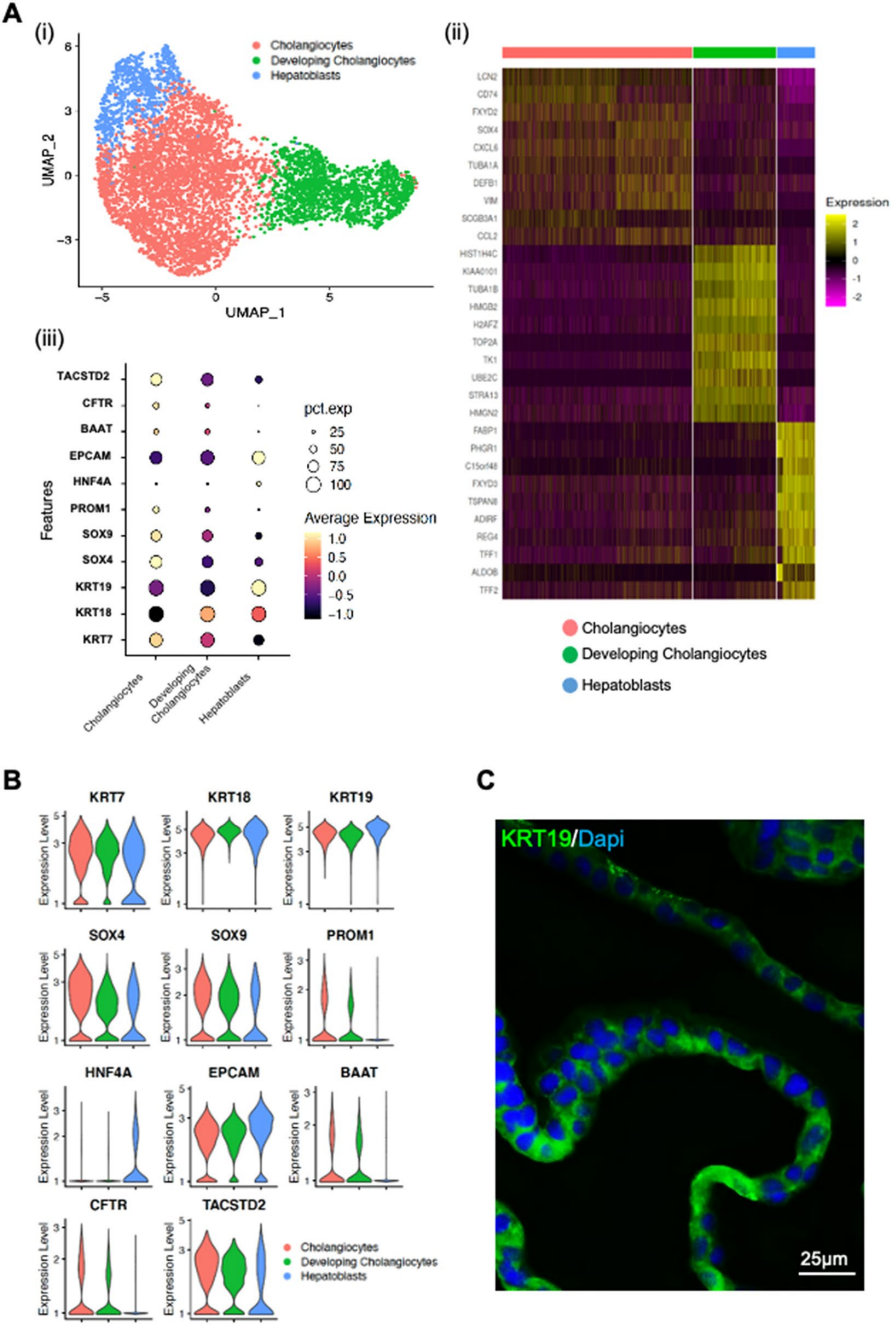
Human liver organoid cells expressed markers of cholangiocyte and hepatoblast. Characterisation of human liver organoids. (**A**(i)) UMAP showing the assigned identity for each cluster (3 clusters) in 10X Genomics single-cell RNA sequencing analysis of human control liver organoids. (**A**(ii)) Heatmap of top 10 markers expressed for each clusters in HB control organoids at *p* < 0.05. (**A**(iii)) Dot plot showing expression of canonical markers used for identification of clusters including cytokeratin-19 (KRT19) and epithelial cellular adhesion molecule (EPCAM). (**B**) Violin plots showing levels of expression of canonical markers for cholangiocytes and hepatoblast used for classification of cell types in control liver organoids. (**C**) Immunofluorescence staining for KRT19 of human control liver organoids.

To investigate if human liver derived organoids can be used to establish an ex vivo SARS-CoV-2 infection model for intra-hepatic bile ducts, we first determined whether the organoid culture could preserve the cholangiocytes expressing ACE2 and TMPRSS2 ex vivo. We performed single-cell RNA sequencing (scRNA-seq) analysis of human liver organoids from 2 patients to interrogate the transcriptomic signatures of cells in human liver organoids. The organoids used in the study are derived from the hepatoblast (intrahepatic ducts) progenitor (KRT19 immuno-positive) lineage, and thus recapitulate the intrahepatic cholangiocyte development. A total number of 5885 cells were analyzed and cell populations were visualized by uniform manifold approximation and projection (UMAP), partitioning the cells into three clusters (Fig. 1A). The common cholangiocyte markers epithelial cell adhesion molecule (EPCAM) and cytokeratin 19 (KRT19) were uniformly highly expressed in all the clusters, indicating the heterogeneity of cholangiocytes in these organoids was relatively low (Fig. 1A, B). Furthermore, immuno-fluorescence staining of the liver organoids with cholangiocyte markers: cytokeratine 19 (KRT19) and γ-glutamyltranspeptidase (GGT); cholangiocyte cell membrane secretin receptor (SCTR), bile acid transporter (ASBT) and chloride channel (CFTR) indicated that cholangiocytes were the major cell type in the liver organoids (Figs. 1C and S1).

**Figure 2:**
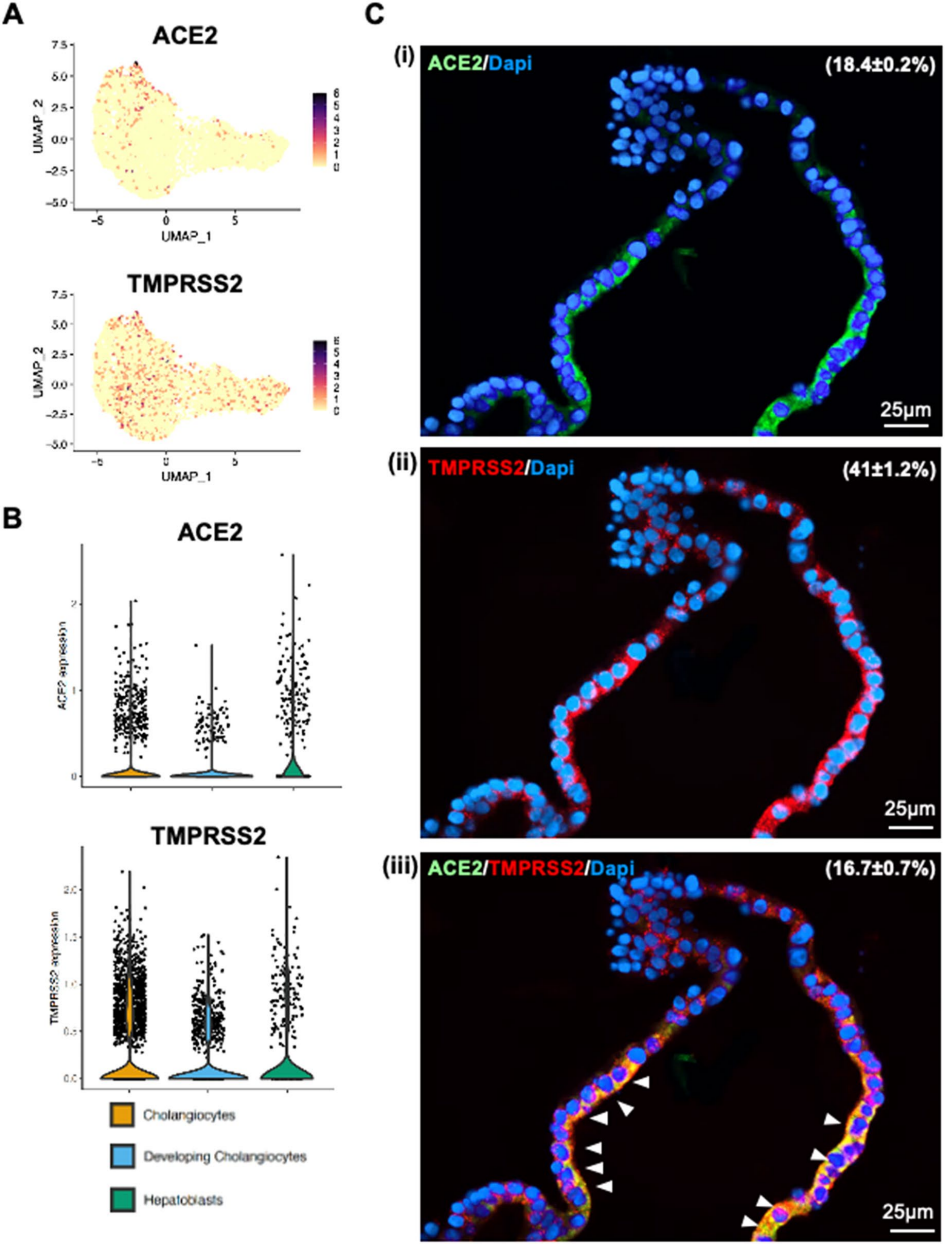
Human liver organoid cells expressed ACE2 and TMPRS2. (**A**) and (**B**) Feature plot and violin plot displaying single cell expression distributions for features (ACE2, TMPRSS2 and TMPRSS4) in each cluster of cells from liver organoids. (**C**) Co-immunofluorescence staining of human liver organoids for ACE2 and TMPRSS2. Parenthesis indicated the percentages of organoid cells that expressed ACE2 (i), TMPRSS2 (ii) and ACE2 plus TMPRSS2 (iii). Arrowheads, organoid cells that co-expressed ACE2 and TMPRSS2.

Notably, we identified the SARS-CoV-2 receptor gene ACE2 expressed sparsely among all the cluster in unbiased preferences and was detectable in 7.32% cells of developing cholangiocytes, 4.78% cells in cholangiocytes and 17.48% cells in hepatoblasts (Fig. 2A and B). Besides, TMPRSS2 expressed uniformly across all the clusters and accounted for 30.18% of developing cholangiocytes, 20.52% in cholangiocytes and 17.89% in hepatoblasts, it is worth mentioning that the ACE2-expressing cells were co-expressing TMPRSS2 (159 out of 233) (Fig. 2A, B), making this cell population potentially highly vulnerable to SARS-CoV-2 infection. Interestingly, these same cells were also found to express another serine protease, TMPRSS4. Co-immunostaining of human liver ductal organoids for ACE2 and TMPRSS2 revealed that nearly all the ACE2-expressing cells also expressed TMPRSS2 (Fig. 2C). Taken together, our data demonstrate that human liver tissue derived organoid preserves the human-specific ACE2-expressing and TMPRSS2-expressing populations of intra-hepatic cholangiocytes.

### Intra-hepatic cholangiocytes are susceptible to SARS-CoV-2 infection

**Figure 3:**
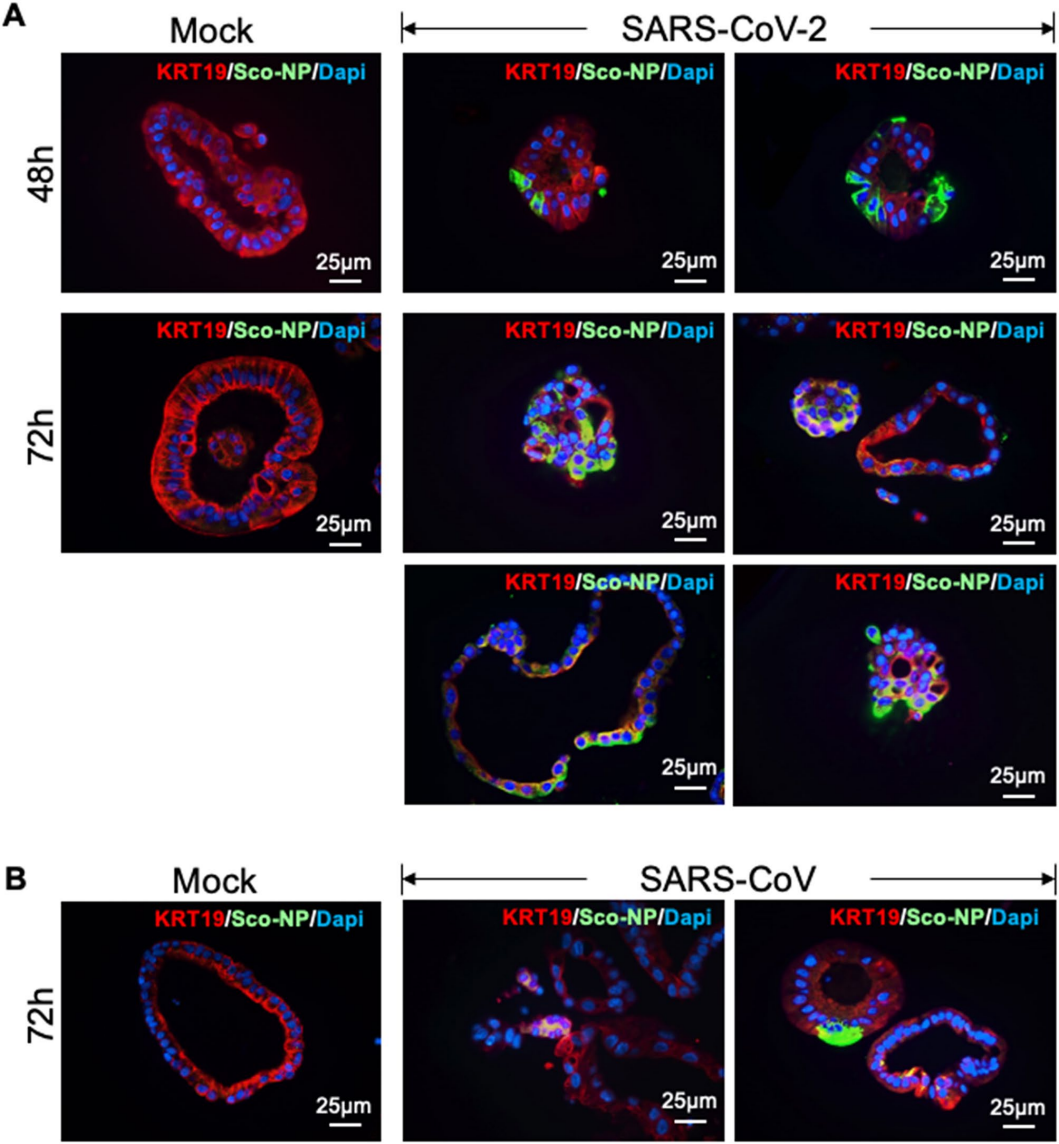
Human liver organoids are susceptible to infection by SARS-CoV-2 and other coronaviruses. (**A**) Immunofluorescence staining for SARS-CoV nucleocapsid protein (Sco-Np) of mock and SARS-CoV-2 infected organoids at 48 h and 72 h post-infection (4 organoids shown). (**B**) Immunofluorescence staining for SARS-CoV nucleocapsid protein (Sco-Np) of mock and SARS-CoV infected organoids at 72 h post-infection.

Next, we examined the susceptibility of human liver ductal organoids to SARS-CoV-2. We infected liver organoids with the SARS-CoV-2, and the SARS-CoV. The liver organoids from two individuals were inoculated with viruses for 1 h, re-embedded in Matrigel and cultured for 48 and 72 h. We performed immunostaining for SARS-CoV nucleocapsid protein (Sco-Np) to identify the virus-positive cholangiocytes 48 h and 72 h post-infection (Fig. 3). The expression of SARS-CoV nucleocapsid protein (Sco-Np) was detected in patchy areas of human liver organoids infected with SARS-CoV-2 and SARS-CoV, whereas no signal was found in uninfected control (Mock, Fig. 3). At 72 h post-infection, both the % infected organoids and the % infected organoid cells/infected organoids were found to be significantly higher in SARS-CoV-2 infected cultures than in SARS-CoV infected cultures (Table 1). In contrast to mock infected organoids, the SARS-CoV-2 infected organoids underwent apoptosis as indicated by the immuno-reactivity for cleaved caspase 3 (Fig. S2), which indicated that SARS-CoV-2 infection induced cytopathogenicity of organoid cells.

**Table 1:** Infection of liver organoids with SARS-CoV-2 and SARS-CoV.

Virus	% Infected organoids	% Infected organoid cells
SARS-CoV	33.3 ± 5.3 (*n* = 180)	17.3 ± 4.7
SARS-CoV-2	62.5 ± 11.5 (*n* = 160) (*p* < 0.05)	28.2 ± 8.1 (*p* = 0.048)

*n* total number of organoids examined.

### Intra-hepatic cholangiocytes supports robust replication of SARS-CoV-2

**Figure 4:**
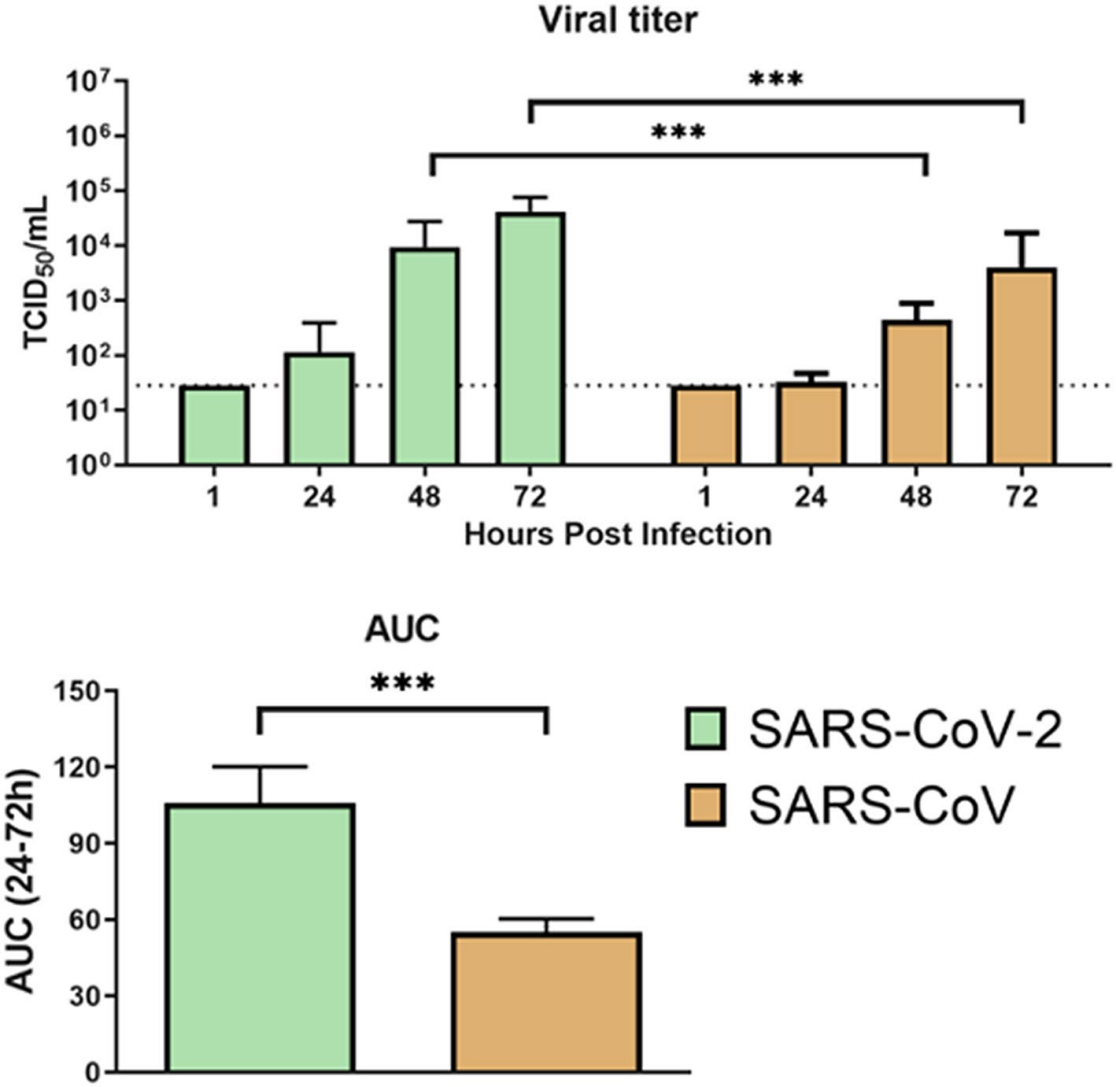
Viral infection and replication kinetics of SARS-CoV-2 and SARS-CoV in human liver organoids. Bar chart showing viral titres in culture supernatants of human liver organoids infected with SARS-CoV-2 and SARS-CoV viruses. The organoids were infected at MOI 0.1 at 37 °C. Culture supernatants were harvested at the indicted times and virus titres were measured by TCID_50_ assay. Bar-charts show the mean virus titre ± standard deviation (SD). The horizontal dotted line denotes the limit of detection in the TCID_50_ assay. Statistical significances compared among viruses were calculated using two-way ANOVA with Tukey’s multiple comparisons test. ***p < 0·001. (C) Bar-charts showing the area under the curve (AUC) derived from the viral titres from 24 to 72 h. Data are presented as mean ± standard deviation (SD). Statistical significances compared among viruses were calculated using one-way ANOVA with Tukey’s multiple comparisons test. ***p < 0·001.

Virus tropism and replication competence of SARS-CoV-2 with SARS-CoV were compared. Here, we showed that the viral titres of SARS-CoV-2 increased starting at 24 h and with more than 2 log increase from 24 to 72 h in bile duct organoids. SARS-CoV-2 replicated significantly higher than that of the SARS-CoV, with about 1 log increase in viral titer along at all-time points (Fig. 4). When comparing the area under the curve (AUC) derived from the viral titers from 24 to 72 h, similar observations were found, in that SARS-CoV-2 had a significantly higher viral replication competence than SARS-CoV (Fig. 4). These data demonstrate that human bile duct organoids are susceptible to SARS-CoV-2 infection and support robust viral replication.

## Discussion

COVID-19, which is caused by SARS-CoV-2, has become a significant global pandemic since January 2020. Although the main route of SARS-CoV‐2 infection remains the respiratory tract, with infected patients displaying symptoms of severe respiratory compromise, presence of virus in the digestive system has been reported and the gastrointestinal tract has therefore been postulated to be another potential portal of viral infection^7–9,24^. The demonstration of abundant ACE2 expression on intestinal cells may suggest a possible direct infection of the gastrointestinal tract by SARS-CoV-2^31,32^. Nonetheless, a clear faecal–oral transmission route for SARS-CoV-2 is still under debate^33^.

In many COVID-19 patients, liver damage, as typified by deranged liver function tests, is seen during SARS-CoV-2 infection^10,11^. The mechanisms of liver injury remain largely undetermined. It has been postulated that direct viral infection, drug cytotoxicity, and a bystander inflammatory immune response may play a role. Given the fact that ACE2 has also been confirmed to be present on liver cells^34^ and that the liver is connected to the gastrointestinal system via the biliary tract, it is indeed a possibility that the liver may also be a potential target for SARS-CoV-2 present in the gastrointestinal tract. Previous report on the detection of SARS‐CoV in liver tissues by RT-PCR provided indirect evidence on the susceptibility of liver cells to SARS-CoV-2 infection^34^. This finding was supported by a recent report which showed the presence of SARS-CoV-2 viral particles in hepatocyte cytoplasm in two COVID-19 patients^23^. Indeed, Zhao et al. recently found that SARS-CoV-2 infection impaired the barrier and bile acid transporting functions of cholangiocytes^27^. In the clinical setting, there has been one case report describing the infection of gall bladder by SARS-CoV-2^35^. the In our study, we further confirmed that human liver organoid derived intra-hepatic bile duct cells provided an excellent ex vivo model for studying SARS-CoV-2 infection. Cholangiocytes were found to co-express both ACE2 and TMPRSS2 and they were highly susceptible to coronavirus infection. In a recent study using an enteroid model, TMPRSS4 protein was shown to be another important serine protease which could work in synergy with TMPRSS2 to release SARS-CoV-2 into the host cell, after viral binding to ACE2^36^. Thus, similar viral interaction and kinetics with intra-hepatic bile duct cells may be expected. Further experiments are underway to determine this.

The three highly pathogenic human coronaviruses include the severe acute respiratory syndrome coronavirus (SARS‐CoV), the Middle East respiratory syndrome coronavirus (MERS‐CoV) and the 2019 new coronavirus (SARS‐CoV‐2). These three viruses can cause diseases in various body systems. Similar to SARS-CoV-2, studies have shown that patients infected with SARS‐CoV and MERS‐CoV can also develop degrees of liver injury. As MERS-CoV uses dipeptidyl peptidase-4 (DPP4) as the spike protein receptor, as opposed to ACE2, we specifically studied and compared only SARS-CoV and SARS-CoV-2 here. Our data demonstrated that SARS-CoV-2 had much higher infectivity and replication rate than SARS-CoV, at least in our intrahepatic bile duct cell organoid system, which is similar to other studies comparing viral tropism and kinetics on respiratory and intestinal cells^37,38^. The higher infectivity of SARS-CoV-2 on tissues would explain why SARS-CoV-2 has caused such a huge worldwide pandemic over the past year. For the ability to infect the liver, previous histological studies showing lobular and portal activity in COVID-19 patients have not pinpointed at-risk cell populations, nor the underlying mechanisms in the course of liver injury^8,9^. Our finding of high susceptibility of intrahepatic cholangiocytes to SARS-CoV-2 infection suggests that biliary cholangiopathy cannot be overlooked both as a cause of liver injury and also as a potential source of viral shedding into the gastrointestinal tract. As COVID-19 pandemic continues to deteriorate, better understanding of SARS-CoV-2 infectivity on different cell populations is important in the development of comprehensive and targeted therapeutic and public health strategies.

## Supplementary Information

Supplementary Information.
