# Supplementary material for: Human liver organoid derived intra-hepatic bile duct cells support SARS-CoV-2 infection and replication
Source: Sci Rep. 2022 Mar 30;12:5375. doi: 10.1038/s41598-022-09306-6 (PMC8965546; doi:10.1038/s41598-022-09306-6)
Supplement: Supplementary file 1 — Supplementary Information. [file 41598_2022_9306_MOESM1_ESM.docx]

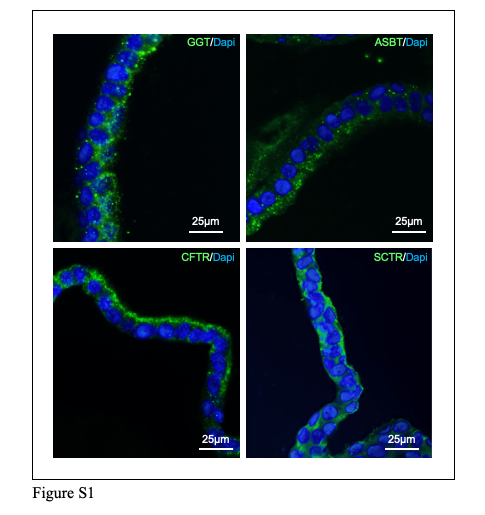


**Figure S1** - Immunofluorescence staining of human liver organoids for GGT, ASBT, SCR and CFTR.


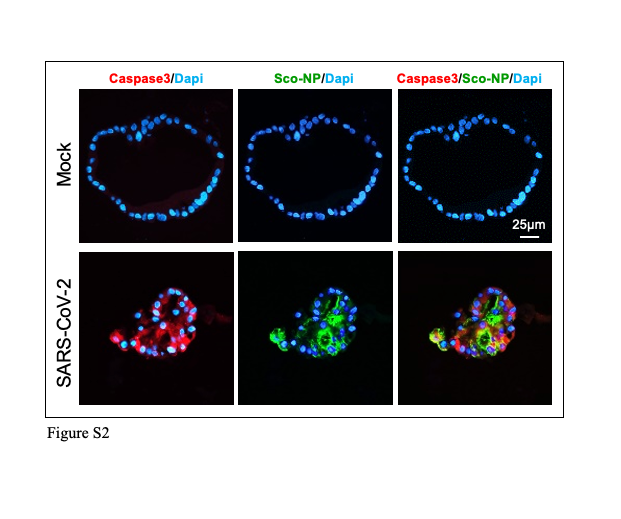


**Figure S2** – Immunofluorescence staining of cleaved Caspase 3 after SARS-CoV-2 infection. Co-immunofluorescence staining for SARS-CoV nucleocapsid protein (Sco-Np; Green) and cleaved Caspase 3 (Red) of mock, and SARS-CoV-2 infected organoids at 72 h post-infection.

| Antigen | Primary antibody | Secondary antibody | Antigen retrieval buffer |
| --- | --- | --- | --- |
| SCov-NP | Anti-Coronavirus nucleocapsid (40143-T62) (Sino Biological; #4013-T62-100); Rabbit polyclonal, (1:1000) | Alexa Fluor^TM^ 488 goat anti-rabbit (Invitrogen; A11008), (1:300) | 10 mM sodium citrate buffer (pH 6.0) at 95°C for 10 min |
| SCov-NP | Anti-Coronavirus nucleocapsid (40143-mm05) (Sino Biological; #4013-T62-100); Mouse monoclonal, (1:1000) | Alexa Fluor^TM^ 594 goat anti-mouse (Invitrogen; A11032), (1:300) | 10 mM sodium citrate buffer (pH 6.0) at 95°C for 10 min |
| ACE2 | ACE2 antibody: Human ACE-2 Alexa Fluor 488-conjugated Antibody (#FAB9332G), (1:10) | Not Applicable | 10 mM sodium citrate buffer (pH 6.0) at 95°C for 10 min |
| TMPRSS2 | Recombinant Anti-TMPRSS2 antibody [EPR3861] (Abcam; ab92323); Rabbit monoclonal, (1:1000) | Alexa Fluor^TM^ 488 goat anti-rabbit (Invitrogen; A11008), (1:300) | 10 mM sodium citrate buffer (pH 6.0) at 95°C for 10 min |
| KRT19 | Anti-Cytokeratin 19 [KRT19/800] (abcam; ab220193); Mouse monoclonal, (1:200) | Alexa Fluor^TM^ 594 goat anti-mouse (Invitrogen; A11032), (1:300) | 10 mM sodium citrate buffer (pH 6.0) at 95°C for 10 min |
| GGT | Anti-GGT1/GGT [EPR5288] (abcam; ab109427); Rabbit monoclonal, (1:100) | Alexa Fluor^TM^ 488 goat anti-rabbit (Invitrogen; A11008), (1:300) | 10 mM Tris Base, 1 mM EDTA, 0.05% Tween 20 (pH 9.0) at 95°C for 10 min |
| SCTR | Anti-Secretin receptor/SCTR (abcam; ab224236); Rabbit polyclonal, (1:200) | Alexa Fluor^TM^ 488 goat anti-rabbit (Invitrogen; A11008), (1:300) | 10 mM sodium citrate buffer (pH 6.0) at 95°C for 10 min |
| CFTR | Anti-CFTR [CFTR/1775R] (abcam; ab219337; Rabbit monoclonal, (1:100) | Alexa Fluor^TM^ 488 goat anti-rabbit (Invitrogen; A11008), (1:300) | 10 mM Tris Base, 1 mM EDTA, 0.05% Tween 20 (pH 9.0) at 95°C for 10 min |
| ABST | Anti-SLC10A2/ABST (abcam; ab203205); Rabbit polyclonal, (1:100) | Alexa Fluor^TM^ 488 goat anti-rabbit (Invitrogen; A11008), (1:300) | 10 mM sodium citrate buffer (pH 6.0) at 95°C for 10 min |
| Cleaved Caspase 3 | Anti-cleaved Caspase-3 (Asp175) (Cell Signaling Technology; #9661); Rabbit polyclonal, (1:400) | Alexa Fluor^TM^ 488 goat anti-rabbit (Invitrogen; A11008), (1:300) | 10 mM sodium citrate buffer (pH 6.0) at 95°C for 10 min |

**Table S1** - Primary and secondary antibodies used in the study.
